# Phage communities in household-related biofilms correlate with bacterial hosts

**DOI:** 10.3389/frmbi.2024.1396560

**Published:** 2024-10-09

**Authors:** Stefanie Huttelmaier, Weitao Shuai, Jack T. Sumner, Erica M. Hartmann

**Affiliations:** ^1^ Department of Civil and Environmental Engineering, McCormick School of Engineering, Northwestern University, Evanston, IL, United States; ^2^ Center for Synthetic Biology, Northwestern University, Evanston, IL, United States; ^3^ Division of Pulmonary and Critical Care Medicine, Department of Medicine, Feinberg School of Medicine, Northwestern University, Chicago, IL, United States

**Keywords:** virome, built environment, biofilm, host-phage interaction, mycobacteria

## Abstract

The average American spends 93% of their time in built environments, almost 70% of that is in their place of residence. Human health and well-being are intrinsically tied to the quality of our personal environments and the microbiomes that populate them. Conversely, the built environment microbiome is seeded, formed, and re-shaped by occupant behavior, cleaning, personal hygiene and food choices, as well as geographic location and variability in infrastructure. Here, we focus on the presence of viruses in household biofilms, specifically in showerheads and on toothbrushes. Bacteriophage, viruses that infect bacteria with high host specificity, have been shown to drive microbial community structure and function through host infection and horizontal gene transfer in environmental systems. Due to the dynamic environment, with extreme temperature changes, periods of wetting/drying and exposure to hygiene/cleaning products, in addition to low biomass and transient nature of indoor microbiomes, we hypothesize that phage host infection in these unique built environments are different from environmental biofilm interactions. We approach the hypothesis using metagenomics, querying 34 toothbrush and 92 showerhead metagenomes. Representative of biofilms in the built environment, these interfaces demonstrate distinct levels of occupant interaction. We identified 22 complete, 232 high quality, and 362 medium quality viral OTUs. Viral community richness correlated with bacterial richness but not Shannon or Simpson indices. Of quality viral OTUs with sufficient coverage (614), 532 were connected with 32 bacterial families, of which only Sphingomonadaceae, Burkholderiaceae, and Caulobacteraceae are found in both toothbrushes and showerheads. Low average nucleotide identity to reference sequences and a high proportion of open reading frames annotated as hypothetical or unknown indicate that these environments harbor many novel and uncharacterized phage. The results of this study reveal the paucity of information available on bacteriophage in indoor environments and indicate a need for more virus-focused methods for DNA extraction and specific sequencing aimed at understanding viral impact on the microbiome in the built environment.

## 1 Introduction

Continuous interactions between humans and the built environment drive reciprocal exposure to and assembly of indoor microbiota (Young et al., 2023; Klepeis et al., 2001). Niches within the built environment continuously accrue microorganisms sourced from human occupants, outdoor environments, or a mixture of the two, and many of these communities may then serve as a source of exposure back to humans (Gilbert and Stephens, 2018). These exposures influence health and disease, including via the transmission of potential pathogens (Maamar et al., 2020). Understanding the community structure and dynamics of the built environment microbiome is key to deciphering its relationship to human health.

Previous studies have shown variations between microbiomes of different human-constructed environments and even between elements of one type of indoor environment (Yooseph et al., 2013). For example, door handles, toothbrushes, and showerheads as elements in the home environment harbor distinct yet often intersecting taxa (Ross and Neufeld, 2015; Zinn et al., 2020). The availability of water is a major driver of community composition, impacting not only which taxa survive in an environment but also their level of activity (Lax et al., 2019). However, even within niches experiencing prolonged periods of wetness, microbiome composition is not uniform. Whether and how human occupants interact with a niche profoundly impacts the proportion of human-associated organisms in the resulting community. For example, surfaces experiencing direct contact with human skin, e.g., touch screens or handles, tend to reflect the human skin microbiome (Hsu et al., 2016).

Studies on built environment microbiomes have largely focused on bacterial members or non-bacterial pathogens, with a few notable exceptions (Ibfelt et al., 2015; Prussin et al., 2019). Despite their importance, research on the roles viruses play in built environment is very limited. In a built environment study sampling 738 metagenomes from residences, subways, and public facilities, 66% (310/471) of recovered viral operational taxonomic units (vOTUs) were found in residences (Du et al., 2023). In another study carried on mass transit systems (MetaSUB), no viruses were identified consistently (in >70% of samples) in 4,728 metagenomes. Results indicated that viral populations correlated with host populations in these environments and that viral communities were distinct between surfaces and air (Du et al., 2023; Mason et al., 2016). As much as the bacterial content of the built environment lacks a common “core,” the viral content seems even more variable. In-depth studies on viromes, especially on bacteriophages, in specific built environments are needed to understand the ecological interactions between viruses and bacteria which shape the built environment microbiota.

As the number of observations and the availability of data increase, quantifying which factors shape the built environment microbiomes and the magnitude of their impact is becoming feasible. Among those factors, availability of water and the degree of human interaction are likely key. Interactions between viruses and hosts and the physical and chemical characteristics of the environment may have important impacts, especially on infrequently detected or less abundant community members. To better understand factors influencing the built environment microbiome in general and the virome in particular, we contrast showerhead and toothbrush microbiomes, as both are characterized by biofilm-based communities that likely harbor virus-host interactions and are frequently wet. However, they differ in their interaction with human occupants: while there is direct contact between toothbrushes and the human oral cavity, showerheads rarely receive any direct human inputs.

Previous studies have shown that showerheads contain both pathogens and antimicrobial resistance genes (Webster et al., 2021; Gebert et al., 2018). In addition, non-tuberculosis mycobacteria were shown to be overabundant in showerheads with a municipal water source. Indeed, water sources were the most important indicator of microbial community composition. In contrast, toothbrush microbiomes contain a mix of human oral-associated and environmentally sourced organisms. No strong associations were found between toothbrush microbiome composition and any available meta-data, including oral hygiene practices and storage location, but the antimicrobial resistance gene diversity was strongly related to the environmentally sourced community members (Blaustein et al., 2021).

The built environment microbiome is highly variable and impacted by a multitude of factors. Understanding the nature and magnitude of these impacts, including the potential role of bacteriophage in governing microbial community structure and function, is essential for informing design that promotes human and environmental health, as well as the longevity of the elements that comprise our buildings. Studying phage and their hosts using a metagenomics approach provides a better understanding of phage-bacteria interactions in biofilms and potentially facilitates biofilm control. This study assessed 96 showerhead samples and 34 toothbrush samples using metagenomic sequencing. Leveraging bioinformatic pipelines designed for virome studies, we identify phages in these environments, study their connections with bacterial communities, and characterize the potential roles they play in shaping their perspective microbiomes as well as affecting health for the humans interacting with these environments.

## 2 Materials and methods

### 2.1 Sample collection, preparation, and sequencing

Both toothbrush and showerhead datasets were collected using community science initiatives. Collection and processing have been previously described in detail by Webster et al., 2021 and Blaustein et al., 2021 for showerheads and toothbrushes respectively (Blaustein et al., 2021; Webster et al., 2021).

Briefly, 496 showerhead biofilms were sampled by volunteers from across the United States and submitted for amplicon sequencing with corresponding metadata. Of these, 92 samples were selected for metagenomic sequencing. Selection of the 92 samples was first based on the non-zero presence of Mycobacterium determined by 16S, and then split evenly between well *versus* public water sources. DNA was extracted and used to build libraries for sequencing on an Illumina HiSeq 4000 at the NUSeq core facility (Northwestern University). Each library was sequenced twice on a different flow cell to produce 184 2x150bp read datasets. Two extraction blanks were also produced and sequenced per flow cell. Technical sequencing duplicate files were concatenated to produce one set of forward and reverse reads per sample for a total of 92 metagenomes and 4 blanks.

The 36 toothbrush samples and corresponding metadata were collected from volunteers within a 100-mile-radius of Northwestern University, Evanston, IL, USA. DNA was extracted and prepped for sequencing on an Illumina HiSeq 4000 at the NUSeq core facility to create 34 metagenomes with 2x150bp reads.

### 2.2 Metagenome data processing and analysis

#### 2.2.1 Pre-processing and metagenomic assembly

Reads were quality filtered and adapter trimmed using fastp (v0.20.1) (optional arguments: “–detect adapter for pe” –length required 50) (Chen et al., 2018). Unpaired reads and reads that did not meet quality cutoff scores were dropped. Cleaned reads were decontaminated by mapping to the Gr38 human reference genome using Bowtie2 (v.2.4.5) and parsed using samtools (v1.10.1) (Langmead and Salzberg, 2012; Danecek et al., 2021). Data before and after quality control were manually assessed using fastqc (v 0.11.9) and multiqc (v1.2)(“Babraham Bioinformatics - FastQC A Quality Control Tool for High Throughput Sequence Data,” n.d; Ewels et al., 2016). Metagenomic sequence diversity and estimated coverage were calculated using Nonpareil 3 (Rodriguez-R et al., 2018).

Reads were assembled on a per-sample basis using metaSPADES (v3.15.5) (Nurk et al., 2017). Assembly quality was checked using Quast (v.5.2.0) (Gurevich et al., 2013). Contigs were binned using Metabat2 (2.12.1), MaxBin2 (v.2.2.7) and Concoct (v.1.0.0) then bins were combined using the MetaWRAP (v.1.3.2) bin refine module (Kang et al., 2015; Wu et al., 2016; Alneberg et al., 2014; Uritskiy et al., 2018). Bin quality was checked using CheckM (v.1.0.12) and bins with greater than 70% completeness and less than 10% contamination were kept for further analysis (Parks et al., 2015). GTDB-tk (v.2.1.1) was used to identify bacterial taxonomy (Chaumeil et al., 2020).

To assess bacterial diversity, short reads were run through MetaPhlAn (v.4.0) on a per sample basis (Blanco-Míguez et al., 2023). Diversity was also assessed using assembly. MAG abundance was determined by aligning reads from each sample to all MAGs using BBMap (v.39.01) with the flag: -ambiguous=best (Bushnell, 2014). To aggregate binned contig statistics into bins, bin contigs were flagged with a bin ID prior to read mapping. After mapping, length and base values were summed on a per bin and per sample bases. Coverage of each bin in sample was determined by dividing the sum of bases by the sum of length.

#### 2.2.2 Metagenomic virus assessment and characterization

Putative phage contigs were identified using VIBRANT (v1.2.1) with default parameters, VirSorter2 (v.2.2.4) with default parameters, and geNomad (v.1.5.2) with default parameters (Kieft et al., 2020; Guo et al., 2021; Camargo et al., 2023). Viral contigs were checked for completeness using CheckV (v.1.0.1) (Nayfach et al., 2021). Alignment of all three viral contig ID outputs was done using megablast. Viral contigs were clustered at 95% nucleotide identity and 85% alignment fraction to create representative vOTUs using the anicalc.py and aniclust.py python scripts from the CheckV GitHub repository. The longest sequence was selected from each cluster as the representative for each vOTU. The vOTUs that were designated as medium quality, high quality and complete by CheckV were kept for downstream analysis.

To determine abundance of vOTUs across samples, cleaned reads from all samples were first aligned to representative vOTUs using BBMap (v.39.01) with the flag: -ambiguous=best (Bushnell, 2014). Metapop (v.0.0.42) was used to create an abundance table (Gregory et al., 2022). Raw abundance was calculated as the average sequencing depth truncated to the central 80% (termed as TAD). Normalized abundance was calculated by scaling the TAD by the number of reads mapped to viral contigs in each sample.

Open reading frames (ORFs) in above-medium quality vOTUs were predicted using Prodigal (v.2.6.3), then taxonomy was assigned using vContact2 (v.0.11.0) (Hyatt et al., 2010; Bin Jang et al., 2019). Phage host predictions were made using iPHoP (v.1.3.2) (Roux et al., 2023). The network created from iPhoP outputs mapped vOTUs to the most likely host based on multiple phage host pairing tools. Further, the iPhoP host database was built from the GTDB database and was customized to add MAGs identified in our samples. MAGs were assigned a taxonomy and placed into the GTDB custom database, which were then paired with sample vOTUs. Viral cluster network and phage host interaction network were visualized using Cytoscape (v.3.9.1) (Shannon et al., 2003). Viral contigs associated with Mycobacterium were searched against the representative virus genomes (ref_viruses_rep_genomes, downloaded from https://ftp.ncbi.nlm.nih.gov/blast/db/ on 2/16/2024) using BLASTn (v2.12.0) (Morgulis et al., 2008; Camacho et al., 2009).

FastANI was used to calculate assembly-wide average nucleotide identity (ANI) of vOTUs connected with Mycobacterium genus (Jain et al., 2018). Output was visualized using Python library Matplotlib. Functions of the predicted ORFs were annotated using EggNOG-mapper (v2.1.12) (Cantalapiedra et al., 2021; Huerta-Cepas et al., 2019). All the ORFs that did not have EggNOG-mapper hit or were not annotated with any functions in categories of Clusters of Orthologous Genes (COG), KEGG KO or BRITE, Carbohydrate-Active enZYmes (CAZy), Pfam, Gene Ontology (GO), or Biochemical Genetic and Genomic (BiGG) databases were denoted as “uncharacterized/hypothetical” ORFs. All ORFs were clustered with 60% coverage and 30% amino acid sequence identity using MMseqs2 (v14.7e284) with a clustering mode that includes protein fragments in the clusters (Steinegger and Söding, 2017). Nucleotide sequences of vOTUs were searched for antibiotic resistance genes using Resistance Gene Identifier (RGI v6.0.2) with the Comprehensive Antibiotic Resistance Database (CARD v3.2.6) (Alcock et al., 2023) and virulence factors using BLASTn (v2.12.0) with the Virulence Factor Database (VFDB) full dataset (Alcock et al., 2023; Liu et al., 2021). Antibiotic resistance gene hits were filtered by MAPQ score ≥ 50, sequence identity ≥ 30%, and percentage length of reference sequence ≥ 80%. Percent alignment to virulence factor were set at ≥ 30% to get counts of loose hits as the average alignment percentage was low.

To construct a phylogenetic tree, Genomad protein annotations were searched for Major Capsid Proteins (MCP). Genes identified as MCP were parsed into an amino acid fasta file, aligned using Muscle (v.5) (Edgar, 2021) with default parameters, and organized into a newick tree using FastTree (v.2.1) (Price et al., 2010). The tree was visualized in R using TreeIO (Wang et al., 2020).

### 2.3 Data visualization and statistical analysis

All downstream analyses were conducted in R, unless otherwise noted. Alpha diversity indexes and Bray-Curtis dissimilarity matrices were calculated within sample type for both the bacterial and viral community using the R package vegan (v.2.6.4) (R Core Team, 2020; Oksanen et al., 2022). Principal coordinates analysis (PCoA) was performed with the dissimilarity matrices to visualize viral and bacterial beta diversity. Permutational multivariate analysis of variance (PERMANOVA) was conducted with Bray-Curtis dissimilarity matrices on collected metadata for different sample types. The Benjamini-Hochberg procedure (also known as false discovery rate (FDR), adjusted *p* value referred to as BH adjusted *p* value hereon) was applied to adjust the *p* values of PERMANOVA results (number of permutations = 9999). In addition, Mantel tests were conducted to measure the Spearman correlations between viral community, bacterial community, and numerical sample metadata matrices.

## 3 Results

### 3.1 Quality and quantity of viral contigs are not determined by sequencing depth

**Figure 1:**
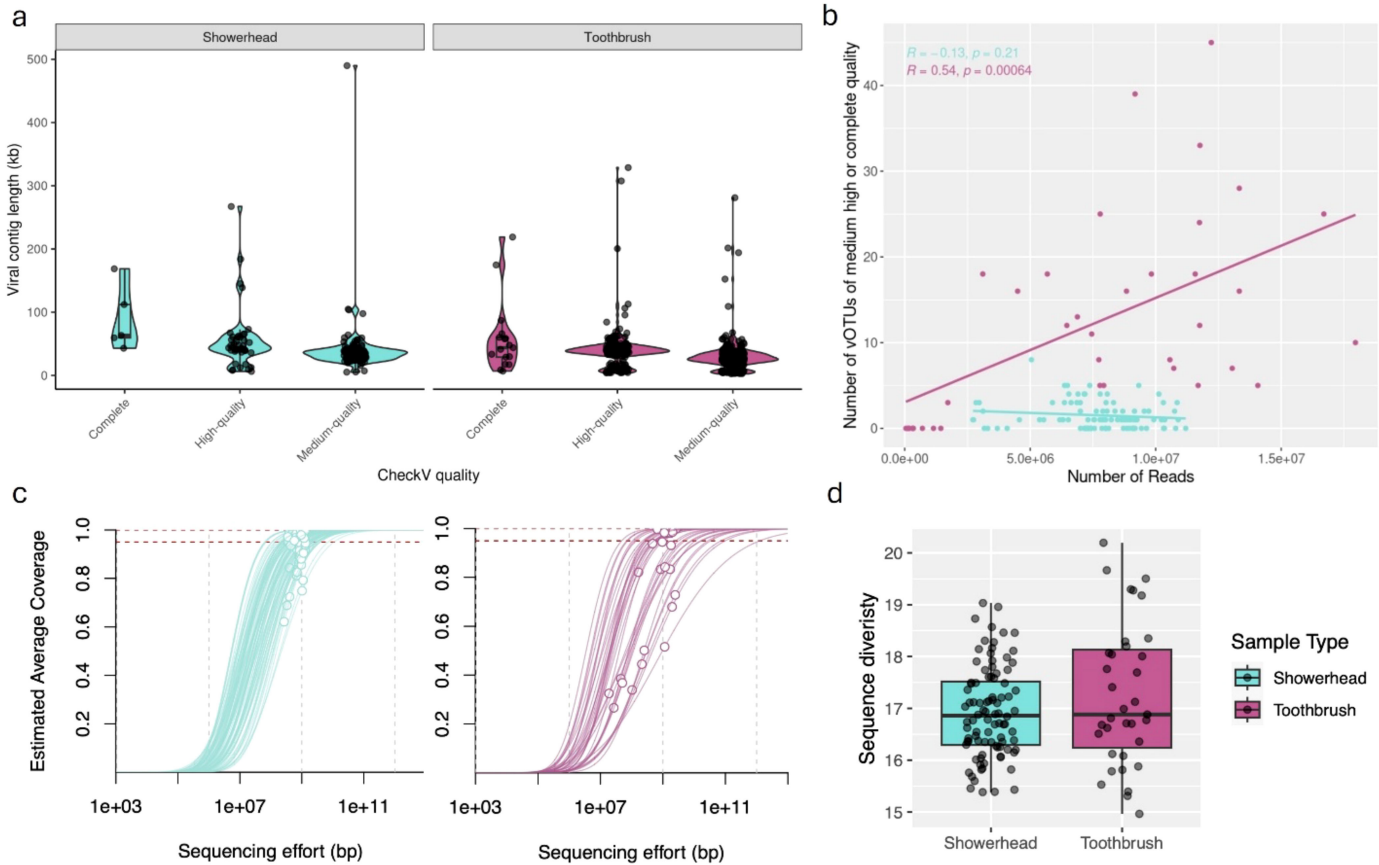
Metagenomes from showerhead and toothbrush show different characteristics. Length of dereplicated, quality filtered viral contigs **(A)**. Number of vOTUs identified from each sample in relation to number of clean reads **(B)**. Nonpareil estimated average coverage, sequencing efforts **(C)**, and sequence diversity **(D)** for each sample.

From 92 showerhead and 36 toothbrush metagenomes, we assembled a total of 72,024,810 scaffolds, of which 1,229,013 were greater than 1 kbp (**Supplementary Table S1**). We identified a total of 8,885, 44,647, and 27,743 viral contigs, using Vibrant, VirSorter2, and geNomad, respectively (**Supplementary Figure S1**, **Table S2**). After combining the three outputs and dereplication, there were a total of 54,358 unique viral contigs, of which 39,503 were greater than 1 kbp in length. Using CheckV, 22 vOTUs were identified as complete (estimated 100% complete), 232 as high quality (estimated >90% complete) and 362 as medium quality (estimated 50-90% complete) for a total of 616 vOTUs from both toothbrush and showerhead samples that were greater than 2.5 kbp in length (**Figure 1A**). Metapop further removed 2 vOTUs, which did not reach a 70% length coverage and 10x mean depth coverage threshold for a total of 614 vOTUs. All other vOTUs were low quality or not determined and were not used in this analysis.

The quantity and quality of viral contigs was not consistent across sample types, even when normalizing for the number of samples and sequencing depth. We consistently have more viral contigs in each toothbrush sample (75% of viral contigs with above-medium quality were identified in toothbrush metagenomes). Toothbrush samples had more reads that passed quality control on a per sample basis, which may have partially contributed to a larger number of above-medium quality viral contigs. Showerhead samples showed much lower counts of above-medium quality viral contigs on a per sample basis compared to toothbrush samples in the same sequence number range (**Figure 1B**), indicating the identification of viral contigs from showerhead samples is likely saturated. The N50 did not impact the number of vOTUs identified in either sample type (showerheads: R = 0.095, *p* = 0.37; toothbrushes: R = 0.18, *p* = 0.3).

In addition to sequencing depth, the abundance of viruses could also artefactually impact our ability to identify viral contigs. We would expect low relative abundance viruses to be less likely to produce reads and thus less likely to be detected and assembled. The number of reads mapped to above-medium quality vOTUs did not impact the number of above-medium quality vOTUs identified in toothbrushes (R = 0.029, *p* = 0.87); however, a correlation was observed in showerheads (R = 0.26, *p* = 0.011).

Toothbrush samples showed lower metagenomic coverage (**Figure 1C**) and higher upper bound and range of metagenomic sequence diversity (**Figure 1D**) compared to showerhead samples, indicating more diverse microbiomes. Under these conditions, we would expect that increased sequencing depth would increase the number and quality of viral contigs. However, our data indicate that this is not the case. The number and quality of viral contigs identified in these datasets is not determined by sequencing depth. To capture more of the viral component of the microbial community, specific enrichment techniques are likely necessary.

### 3.2 Viral populations in showerhead and toothbrush microbiomes are distinct

**Figure 2:**
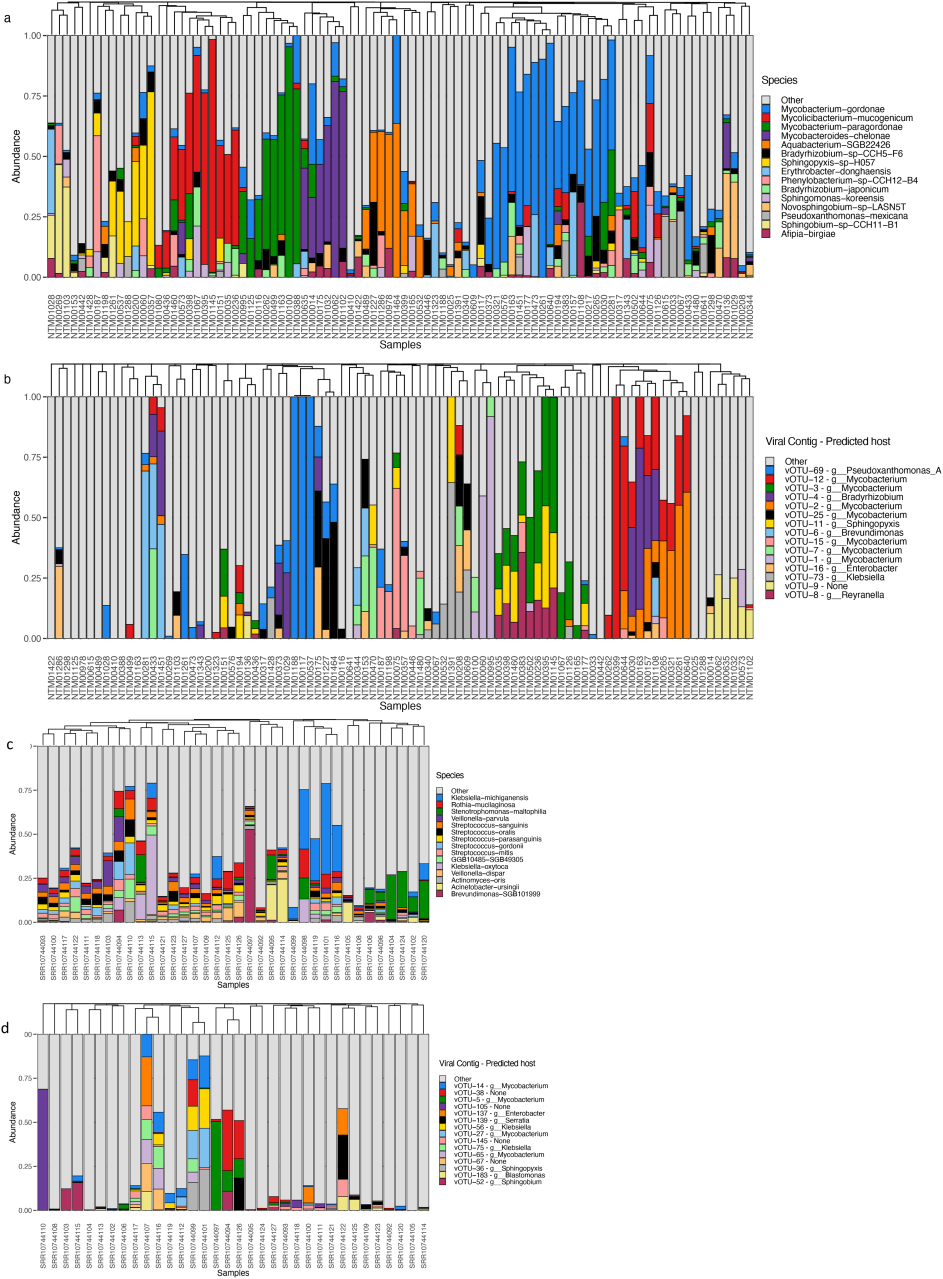
Bacterial **(A, C)** and viral **(B, D)** community relative abundances in showerhead **(A, B)** and toothbrush **(C, D)** samples.

Both showerheads and toothbrushes receive tap water as input for the microbial community, thus we may expect some overlap in community composition (**Figures 2A, C**). We compared relative abundances of top ranked bacterial taxa and normalized abundance of top ranked viral taxa across both sample types (**Figures 2B, D**). Of 614 vOTUs, 314 were detected in only one sample and no vOTU was shared across all 126 metagenomes. Viral taxa featured low average relative abundance with low frequency appearing in both showerhead and toothbrush samples (**Supplementary Figure S2**), although some vOTUs had high relative abundance in several microbiomes (**Figures 2B, D**). This feature of frequency-abundance relationship differed from that of bacterial community on toothbrushes (Blaustein et al., 2021), indicating that there might not be a core group of viral taxa that characterizes the niche environment viromes. There was no overlap in the top 15 most abundant vOTUs in showerhead and toothbrush samples, indicating distinct viral populations exist in the two different types of household biofilms. This discrepancy reflects the overwhelming contribution of the human microbiome to toothbrushes: with the exception of *Brevundimonas*, all of the most abundant bacterial taxa detected on toothbrushes are commonly associated with humans, primarily in the oral cavity (**Figure 2C**). In our previous comparison of the bacterial communities, only *Pseudomonas* and *Stenotrophomonas* were found in both sample types, with both taxa being more frequently detected on toothbrushes (Blaustein et al., 2021). Nevertheless, for the 154 vOTUs for which major coat protein sequences could be predicted, the phylogenetic distribution was split across both sources, rather than clustered by source (**Supplementary Figure S3**).

### 3.3 Apparent connections between viral and bacterial communities

**Figure 3:**
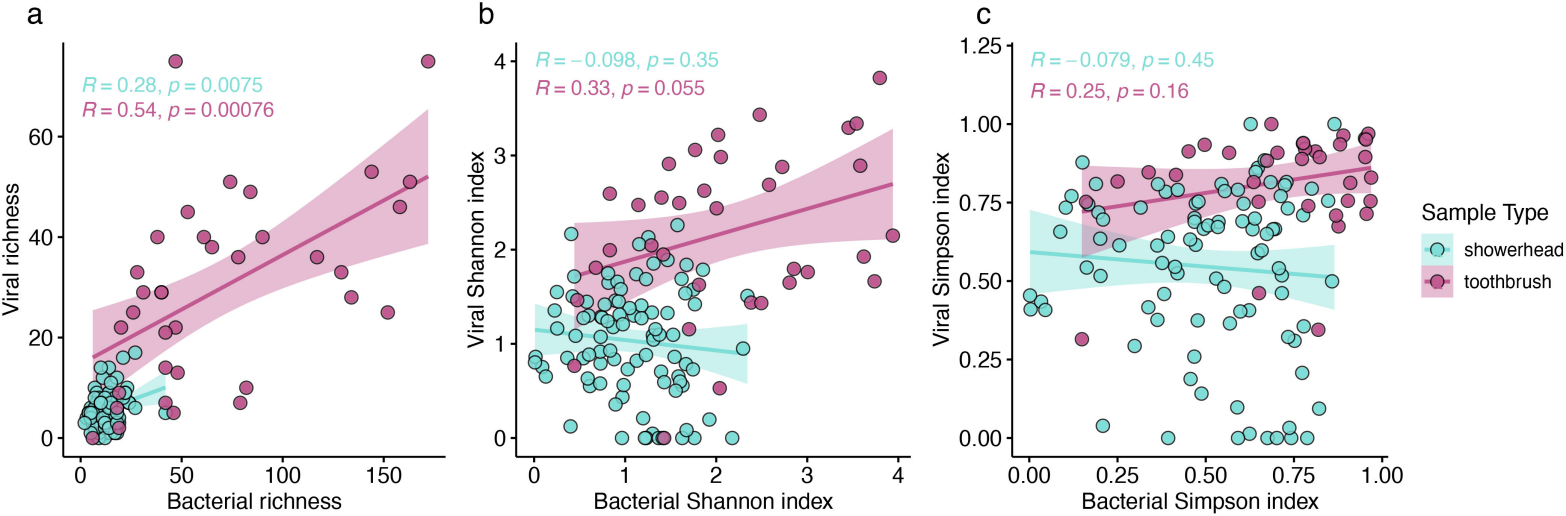
Richness **(A)**, Shannon index **(B)**, and Simpson index **(C)** of viral and bacterial communities. Pearson correlation coefficients and *p*-values were calculated for showerhead samples only (blue), toothbrush samples only (purple).

We hypothesized that more diverse bacterial communities would harbor more diverse bacteriophages. Positive correlations (Pearson correlation, *p* < 0.05) were observed for the richness of viral and bacterial communities in both showerhead and toothbrush microbiomes, but not for Shannon or Simpson indexes, both of which take evenness into account (**Figure 3**). Thus, while a greater number of hosts translates to a greater number of viruses in a community, the evenness of the host distribution is not imparted onto its viral counterpart.

**Figure 4:**
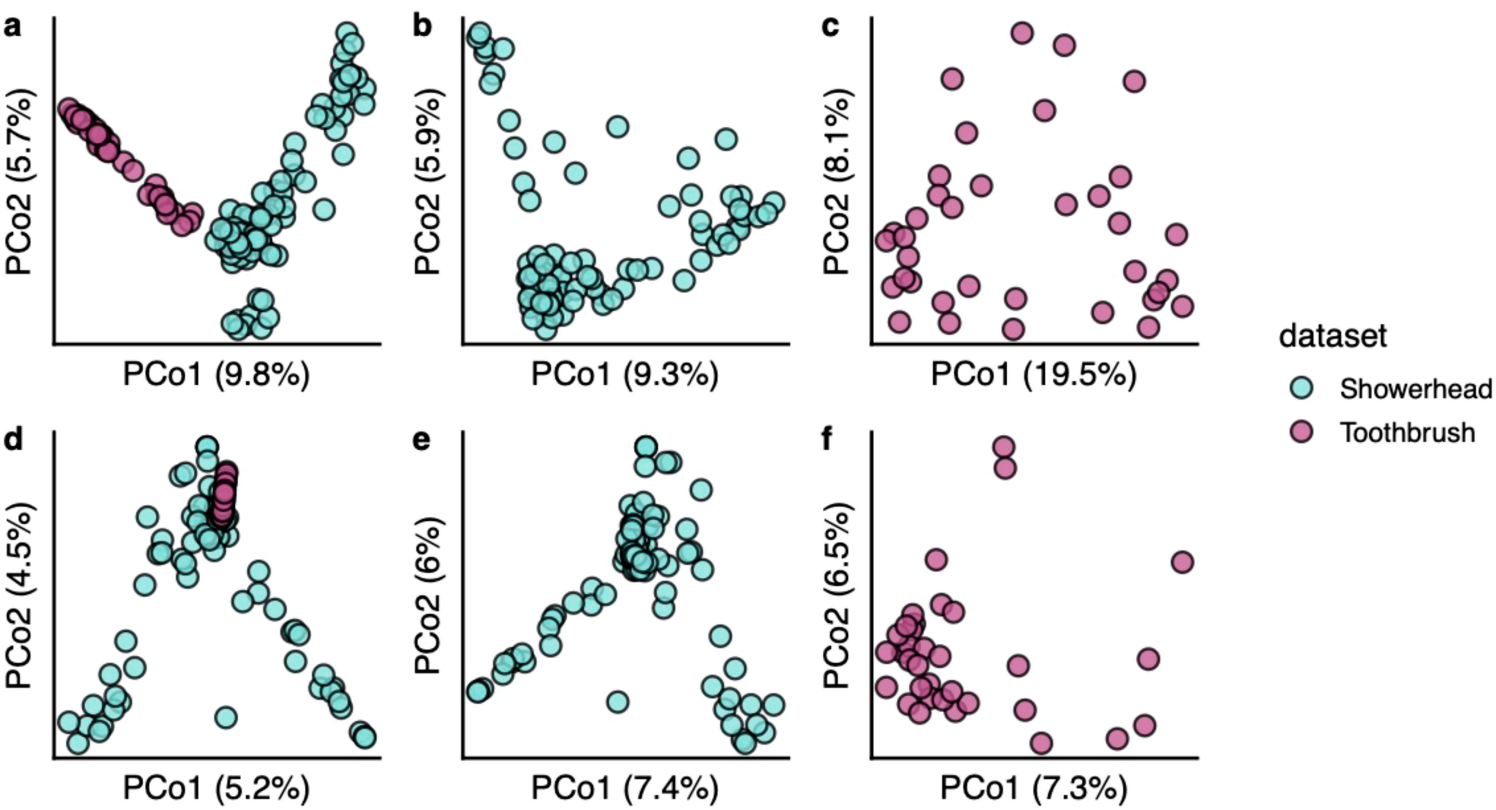
Beta diversity of bacterial communities **(A–C)** and viral communities **(D–F)**. Bray-Curtis distances calculated from arcsine square root transformed relative abundances were used for ordinations.

The toothbrush viral community is not as dispersed as the showerhead viral community (**Figures 4A–C**). There were 16 showerhead samples and 3 toothbrush samples containing singleton vOTUs (defined here as vOTUs found only in one sample). Bacterial communities also showed a similar trend of higher sparsity in showerhead samples (**Figures 4D–F**). This could be result of the geographical distribution of the samples, as the showerhead samples were taken nationwide of the United States while the toothbrush samples were taken within 100 miles of Northwestern University. In addition, the two sample types represent very different environments where showerheads were nutrient limited, and toothbrushes contacted human-related microbiomes, food residues and chemicals.

Among the showerhead sample metadata (**Supplementary Table S3**), only the source of household water was shown as a significant but very weak predictor of the difference in showerhead viral community composition (PERMANOVA R^2^ = 0.026, BH adjusted *p* < 0.01). Metadata collected along with toothbrush microbiomes are all categorical factors, none was significantly associated with the toothbrush viral community composition (PERMANOVA, BH adjusted *p* > 0.05). Similar results were observed for the marker genes based bacterial community profiles, where the only significant but weak association was between the source of household water and the showerhead bacterial community (PERMANOVA R^2^ = 0.046, BH adjusted *p* < 0.01) in our datasets. The previous study on toothbrush microbiomes also showed very weak effects of biotic and abiotic factors shaping the bacterial community composition (Blaustein et al., 2021). The previous showerhead microbiome study that recruited more samples showed that location, climate, water chemistry, water supply and source, and household variables had weak effects (with less than 2% of the variation explained) on the bacterial community composition (Webster et al., 2021). Although documented environmental factors showed minimal to no impact on both viral and bacterial communities in our sample sets, the bacterial community composition had a significant correlation with the viral community composition in both showerhead and toothbrush environments (Mantel statistics r = 0.302 and 0.560 for showerhead and toothbrush, respectively; *p* = 0.001 for both environments).

### 3.4 Genomic evidence of host-phage interactions

**Figure 5:**
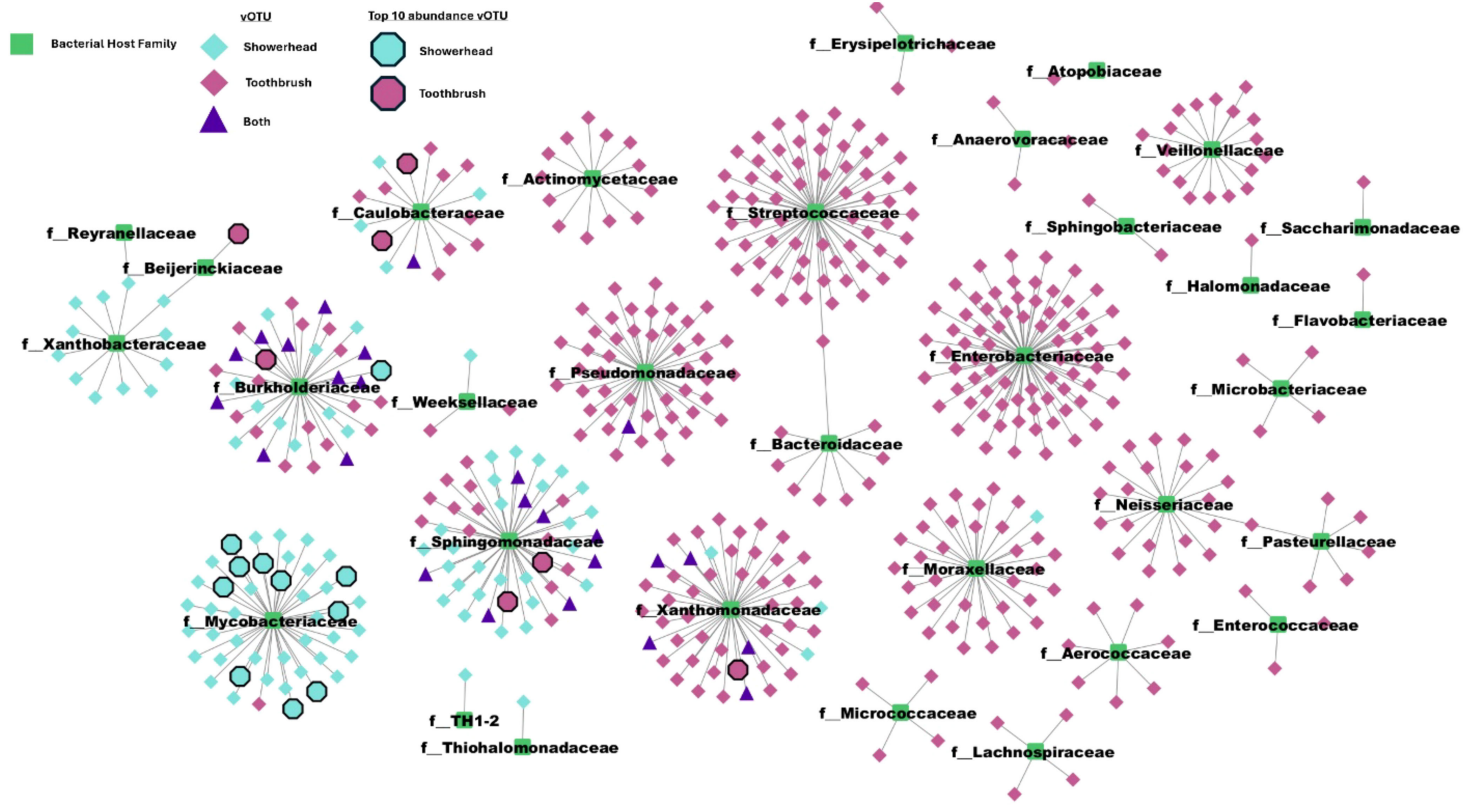
Phage-host network reveals that while most interactions are predominantly specific to a single environment (showerhead or toothbrush), Sphingomonadaceae, Burkholderiaceae, and Caulobacteraceae are identified in common. Center nodes are bacterial taxa from GTDB plus the MAGs recovered from our metagenomes collapsed to family level.

Of 614 quality vOTUs, 532 vOTUs were predicted to associate with hosts in 32 bacterial families. All 32 families contained taxonomy-assigned MAGs in either a showerhead or toothbrush sample. The aggregate phage-host network showed a clear split between sample types; most vOTU-bacterial family pairs appeared in either showerhead or toothbrush microbiomes. Both environments harbor some vOTUs that are associated with Burkholderiaceae, Caulobacteraceae, Pseudomonadaceae, Sphingomonadaceae, and Xanthomonadaceae (purple triangles in **Figure 5**), which are bacterial families identified in both environments with high relative abundances (**Supplementary Table S4**) except for Pseudomonadaceae.

**Figure 6:**
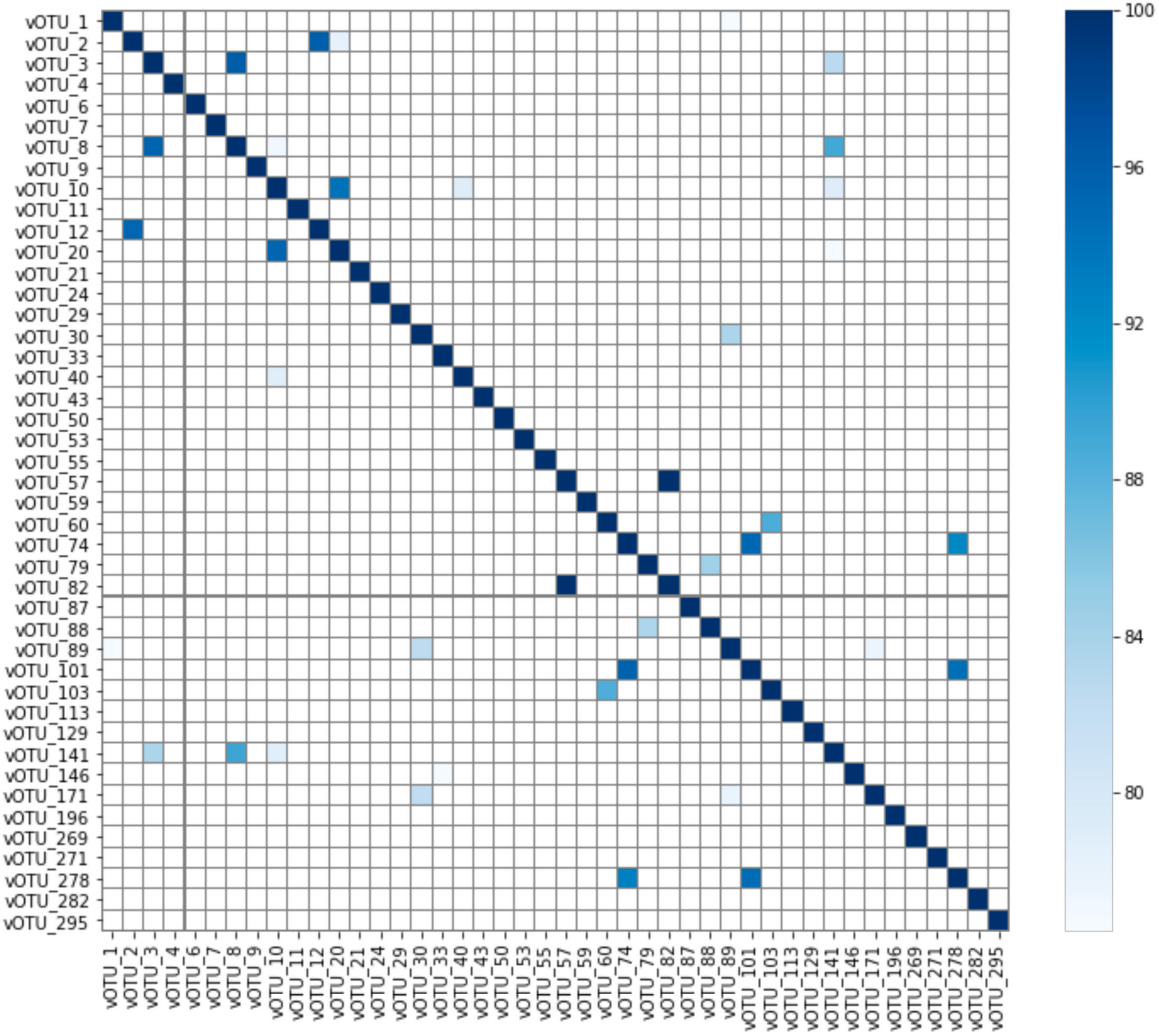
Average nucleotide identity (ANI) of vOTUs connected to genus Mycobacterium. Note that ANI much below 80% will not be reported by FastANI.

As the showerhead samples in this study were selected for those with the presence of *Mycobacterium*, it is not surprising that 44 vOTUs were found to connect with genus Mycobacterium. Although the vOTUs were dereplicated with 95% nucleotide identity and 85% alignment fraction, similarities among the mycobacteriophages were still expected to some degree. However, no clusters were observed based on the average nucleotide identity (**Figure 6**), meaning the mycobacteriophages found in this study, even from similar niche environments (showerheads), possess high diversity in their genomic contents. BLAST against the representative virus database (ref_viruses_rep_genomes) showed that only 19 out of 44 vOTUs associated with *Mycobacterium* yielded hits with > 1 kbp alignment length, and all had less than 85% identity to the database *Mycobacterium* phage sequences (**Supplementary Table S5**). This indicates that novel mycobacteriophages might have been recovered from the metagenomes of showerhead and toothbrush samples.

Zooming in the phage-host network analysis at the MAG level, most of the mycobacteriophage vOTUs were interlinked with multiple *Mycobacterium* MAGs (**Supplementary Figure S4A**). The vOTU with the highest truncated average depth (TAD) among all samples (vOTU_1) is specifically paired with a MAG recovered from the showerhead sample (NTM00995) where vOTU_1 has the highest TAD (**Supplementary Figure S3B**), indicating potential active infection in that microbiome. The best BLAST hit of vOTU_1 is *Mycobacterium* phage IdentityCrisis, whose host is *Mycobacterium smegmatis* mc²155 according to The Actinobacteriophage Database (https://phagesdb.org/phages/IdentityCrisis/).

### 3.5 General functional content of phage-related contigs

**Figure 7:**
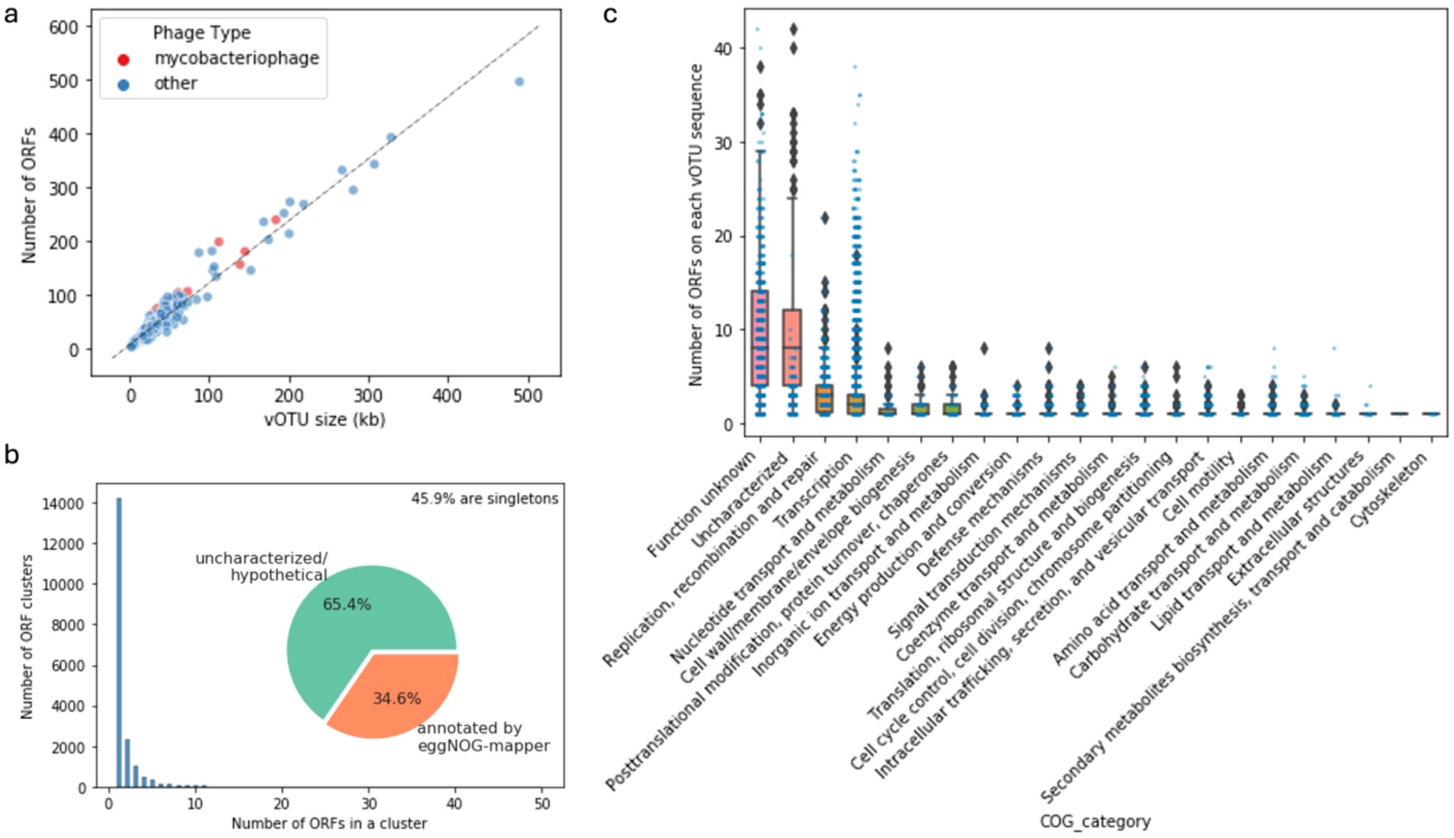
Statistics and characteristics of open reading frames (ORFs) in viral contigs. Number of ORFs and vOTU sequence size **(A)**. Distribution of the sizes of the ORF clusters at 30% amino acid similarity and 60% coverage and the proportions of annotated ORFs **(B)**. Number of ORFs classified in different Clusters of Orthologous Genes (COG) categories per vOTU sequence **(C)**.

There was a large span of vOTU sizes and predicted ORFs in each vOTU sequence, including the group of potential mycobacteriophages identified from our samples (**Figure 7A**). This mirrors the remarkable diversity of mycobacteriophage reported by other studies (Hatfull, 2022). A considerable portion (45.9%) of the ORFs found in the vOTU sequences were singletons based on relatively generous thresholds of 30% amino acid sequence identity and 60% coverage, which highlights the diversity of gene contents of viral communities (**Figure 7B**). The general functional content of the phage-related contigs featured ORFs of uncharacterized/hypothetical proteins and proteins falling into the “function unknown” category of the clusters of orthologous genes (COG, **Figure 7C**). ORFs categorized for functions like replication, recombination and repair, transcription, and nucleotide transport and metabolism were abundant in the vOTUs, which is expected for viruses. Searching the ORFs against the databases of antibiotic resistance and virulence factors did not result in many hits with confidence (**Supplementary Figure S5**), indicating these viruses are unlikely to carry cargo with known adverse human health effects. However, this result further highlights the highly uncharacterized and diverse features of the viral contigs recovered from our samples.

## 4 Discussion

### 4.1 The built environment microbiome

The built environment can refer to several different types of human constructed and occupied environments, from more private spaces like our homes and offices, to less private settings like public transportation or other public use spaces. Each of these distinct settings is further defined by a collection of niche environments that are subject to varying conditions of sunlight, water, chemical input and human interaction.

In our current study, toothbrushes and shower heads look nothing like each other and represent very different niches. They are, nevertheless, both biofilm-dominated engineered environments that happen to be found inside of buildings and that have important implications for human exposure. An evident characteristic of the virome in both showerhead and toothbrush environments is the lack of shared community members, which is not only observed between sample types (**Figure 4A**), but also between different samples within a niche: the abundance heatmap of all above-medium quality vOTUs showed that even viromes of the same sample type do not share many taxa (**Supplementary Figure S6**). This trend is different for bacterial communities, where more similar patterns were observed across samples of the same type (**Supplementary Figure S7**). With bacterial taxonomy assignments, one can observe clearly that the toothbrush environments feature human microbiome related genera such as *Klebsiella*, *Streptococcus*, and *Veillonella* (top 3 ranked genera in toothbrush samples), whereas showerhead environments feature both genera demarcated from *Mycobacterium* (that is, *Mycolicibacterium* and *Mycobacteroides)* and genera commonly found in soil or drinking water (*Sphingopyxis*, *Sphingobium*, and *Aquabacterium*). Similar niches in different built environments may select for similar communities to some extent, but from one built environment to another, the detailed features of the microbial assemblages are likely determined by the impacts of environmental factors at each specific built environment. All this to say, the built environment microbiome is not a monolith.

### 4.2 Impacts of environmental factors

A possible explanation for the lower alpha diversity of showerhead viromes is that showerheads receive very limited inputs (only receiving household water, and the input source is relatively stable) compared to toothbrushes, and the showerhead bacteria communities were hosts of less diverse, less well-known communities of phages. The insignificant effects for most of the environmental factors on viral community could reflect a relatively small sample size in addition to the high variation across the microbiomes in indoor environments. Since the correlations between bacterial and viral community composition matrices were significant, and the source of household water had a much weaker effect on the viral community than on the bacterial community, it is possible that bacterial communities were affected by environmental factors and then modulated the phage communities as their host organisms.

### 4.3 Implications of the host-phage interactions

While we were able to construct a vOTU-bacterium interaction network from metagenomes of the built environment niches, longitudinal sampling would be needed for elucidating the dynamics of the host-phage interactions in these niches. From our snapshot of the host-phage networks from built environment microbiomes, clusters of viral contigs associated with bacterial families contain potential human pathogens, calling for the attention on the implications of the viruses on built environment microbiomes and human health. As these viruses seem unlikely to carry antibiotic resistance or virulence genes as cargo, the viruses themselves may not be a high priority for concern. Conversely, they may be an interesting source of phages for therapeutic application (Stachler et al., 2021).

As the niche environments in this study are generally nutrient limited, we do not expect viral contig ORFs encoding functions related to carbon cycle and nutrient removal to have high abundance as is observed in wastewater treatment viral contigs (Y. Chen et al., 2021). Although higher abundance of antimicrobial resistance genes in the phage DNA fraction compared to bacterial DNA fraction (Subirats et al., 2016) and significant relationship between the profiles bacterial/phage-comediated antimicrobial resistance genes (Yang et al., 2021) were reported in wastewater treatment systems, our study only showed a few instances of antimicrobial resistance in viral contigs recovered from the built environment metagenomes. It is possible that the highly diverse genomic content of the built environment viruses hinders our ability to identify known antimicrobial resistance genes. Whether interactions between phages and their host have noteworthy implications on human health risks such as antimicrobial resistance dissemination in the built environments requires further investigation.

### 4.4 Confounding variables

Technical artefacts, including sample collection and processing and specific sequencing method, are well-known confounding factors for microbiome studies (Adams et al., 2015). Similarly, challenges in sampling low biomass environments and the technical difficulties of producing enough uncontaminated DNA for analysis are well documented for studies focusing on bacteria (Shen et al., 2021). These challenges are exacerbated for viruses, particularly because adsorption of bacteriophages on polypropylene labware affects the reproducibility of phage research (Richter et al., 2021). Although sequencing depth was not observed to impact our ability to identify viruses within a sample type in this study, the quality of sequencing and assembly overall likely influences presence/absence of viruses between different sample types within the built environment. However, sequencing depth alone is insufficient to fully reveal the viral community. In existing metagenomic studies in the built environment generated to query the bacterial community, we are likely only capturing a fraction of the existing virome. Differences in signal to noise ratio of virus to bacterial hosts might impact the number of low signal viruses detected, viral contig assembly, and ultimately the number and quality of viral contigs identified (Kosmopoulos et al., n.d.).

Even in the absence of artefacts, identifying viruses from metagenomes is limited by database bias. For environments like toothbrushes, many of the viruses identified are linked with human-associated bacterial hosts. While this is to be expected, it is unclear whether the environmental contribution, e.g., from tap water, is underestimated or if unknown viruses escape detection due to their lesser degree of documentation. When considering the showerheads, Mycobacteriophages have the highest representation in RefSeq, and are therefore easier to identify with higher certainty (Hatfull, 2020). It is thus unsurprising that we were able to recover many Mycobacterium related phages, but they may be overrepresented because we know to look for them and our tools will identify them. Moreover, viral metagenomic literature generated before 2022 uses morphology-based taxonomy families, *Podoviridae*, *Myoviridae* and *Siphoviridae* to describe the tailed phage community (Turner et al., 2023). As the viral bioinformatic field continues to grow dramatically and viral taxonomy continues to develop, datasets need to be re-analyzed to confirm prior results and facilitate ongoing comparisons.

Linking viral communities to metadata also presents a challenge. Different niches and different studies prioritize different types of metadata, making statistical analysis between studies impossible in some cases. Given the diversity of niches within the built environment, it is perhaps unrealistic to expect harmonization of metadata. The MixS-BE standards include metadata considered to be important for interpreting built environment microbiome results, e.g., the number of occupants in a building. However, it is unclear how relevant that or other prescribed metadata might be to the microbial community within a showerhead or on a toothbrush (Glass et al., 2014). The integration of categorial data, such as material types, with numerical results is a further statistical challenge.

Finally, one important question that we cannot answer with these data is how these environments are changing over time. Phage host interactions are dynamic, and even with tools that allow us to estimate whether a phage will infect a certain host, longitudinal or manipulative studies are needed to corroborate actual infection. In the case of showerheads and toothbrushes, viruses may be transient within the community. The genetic content of viruses, and whether they are transient in a system might inform how stable these communities are, and how vulnerable they might be to change. These challenges all highlight the continued need for expanded method development, longitudinal sampling, and virus-specific analyses to further probe the role of these incredibly diverse entities in the microbial communities that surround us.

## 5 Conclusion

We constructed the network of viral contigs and their potential bacterial hosts from showerhead and toothbrush microbiomes. Although the two niche environments are both in bathrooms of households, the microbiomes, especially the viral communities, are distinct with unique features. These observations suggest that there is little communication between these compartments within the built environment. Viral composition and abundance in the built environment appear not to be directly affected by environmental factors but may be modulated by their bacterial hosts’ response to environmental factors. High disparity and genomic content diversities are the dominant characteristics of the viromes in this current study. No evidence shows risks of viral contigs carrying antibiotic resistance genes or virulence factors in these built environments, but the high diversity of phage taxa and functional genes merits further study to elucidate their implications on human health or utility for biotechnology or therapeutics. With a limited number of built environment metagenomes to compare to, a future study might compare the built environment viral community with natural environments or other engineered environments like wastewater, which have benefited from larger and more robust studies.

## Data Availability

Publicly available datasets were analyzed in this study. This data can be found here: https://www.mg-rast.org/mgmain.html?mgpage=project&project=mgp87891, https://www.ncbi.nlm.nih.gov/bioproject/596937.
