# Supplementary material for: Phage communities in household-related biofilms correlate with bacterial hosts
Source: Front Microbiomes. 2024 Oct 9;3:1396560. doi: 10.3389/frmbi.2024.1396560 (PMC12993545; doi:10.3389/frmbi.2024.1396560)
Supplement: Supplementary file 1 [file DataSheet1.docx]

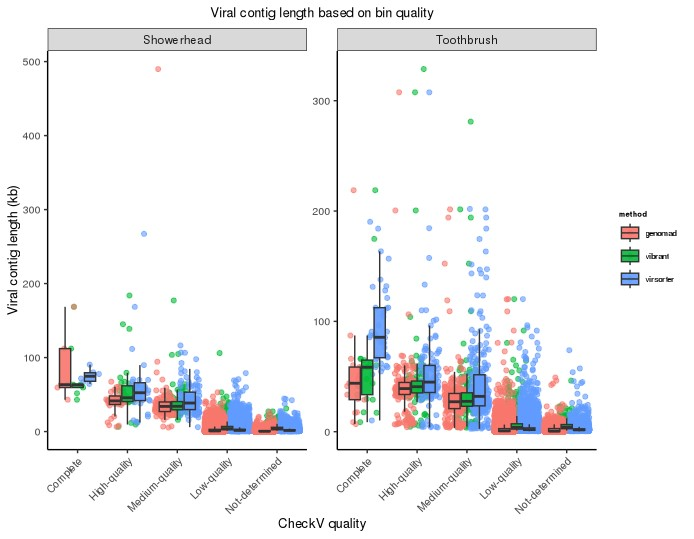

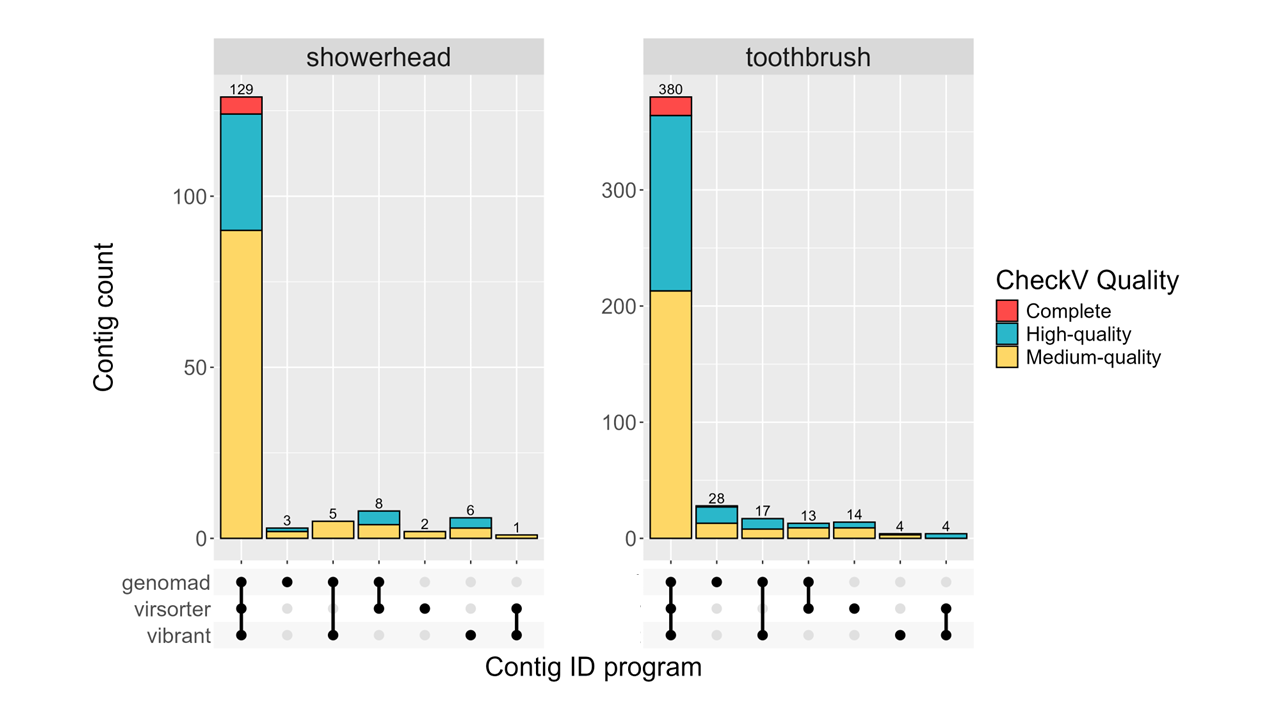

**Fig. S1 (a)** All viral contigs identified by three different viral identification programs, by length and quality. **(b)** The vOTUs kept after dereplication, and which programs they were initially identified with.


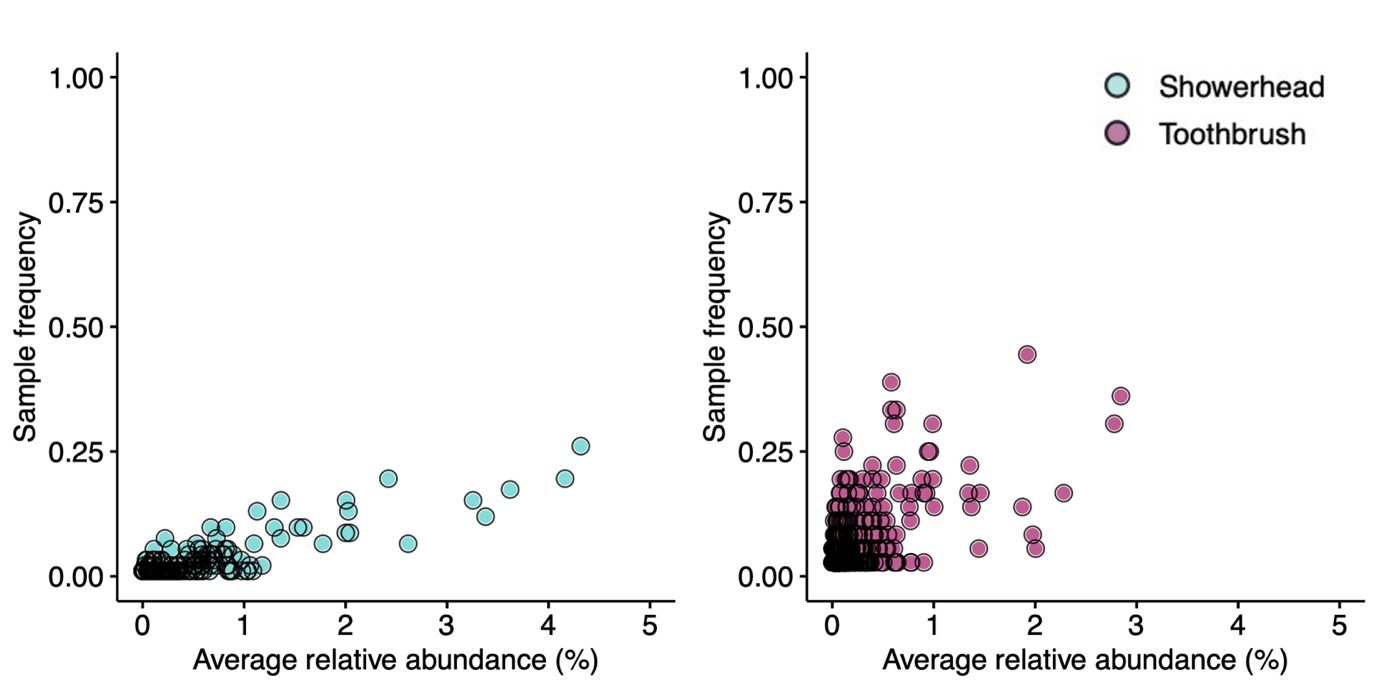


**Fig. S2** Frequency-abundance of showerhead and toothbrush viral communities.

**Fig. S3** Phylogenetic relatedness of vOTUs based on the predicted major capsid protein sequence. Tips are labeled by the source of the vOTU.


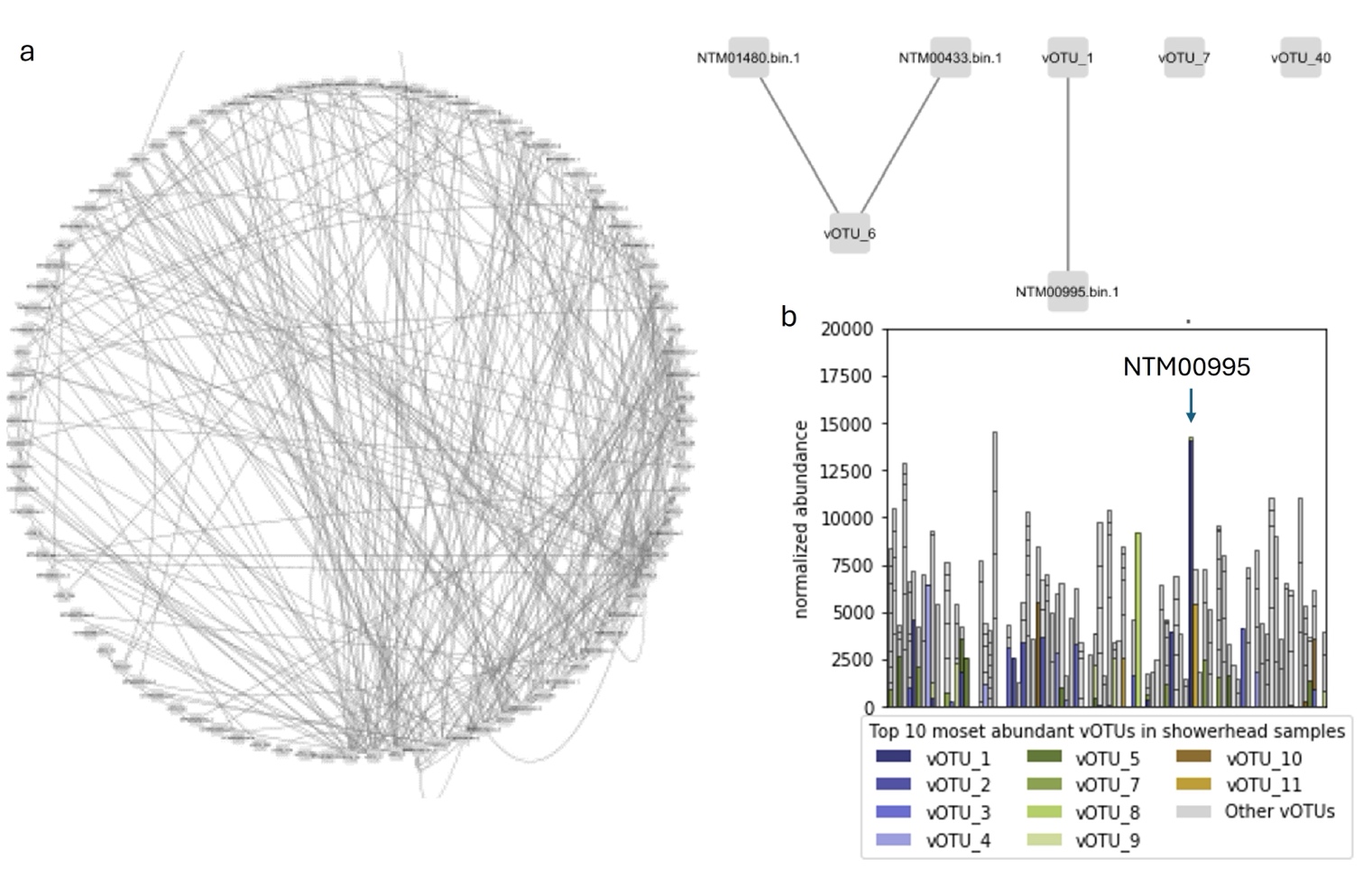

**Fig. S4** Mycobacteriophage vOTU-MAG network (a) and normalized abundance of vOTUs in showerhead samples shown in truncated average depth (b).


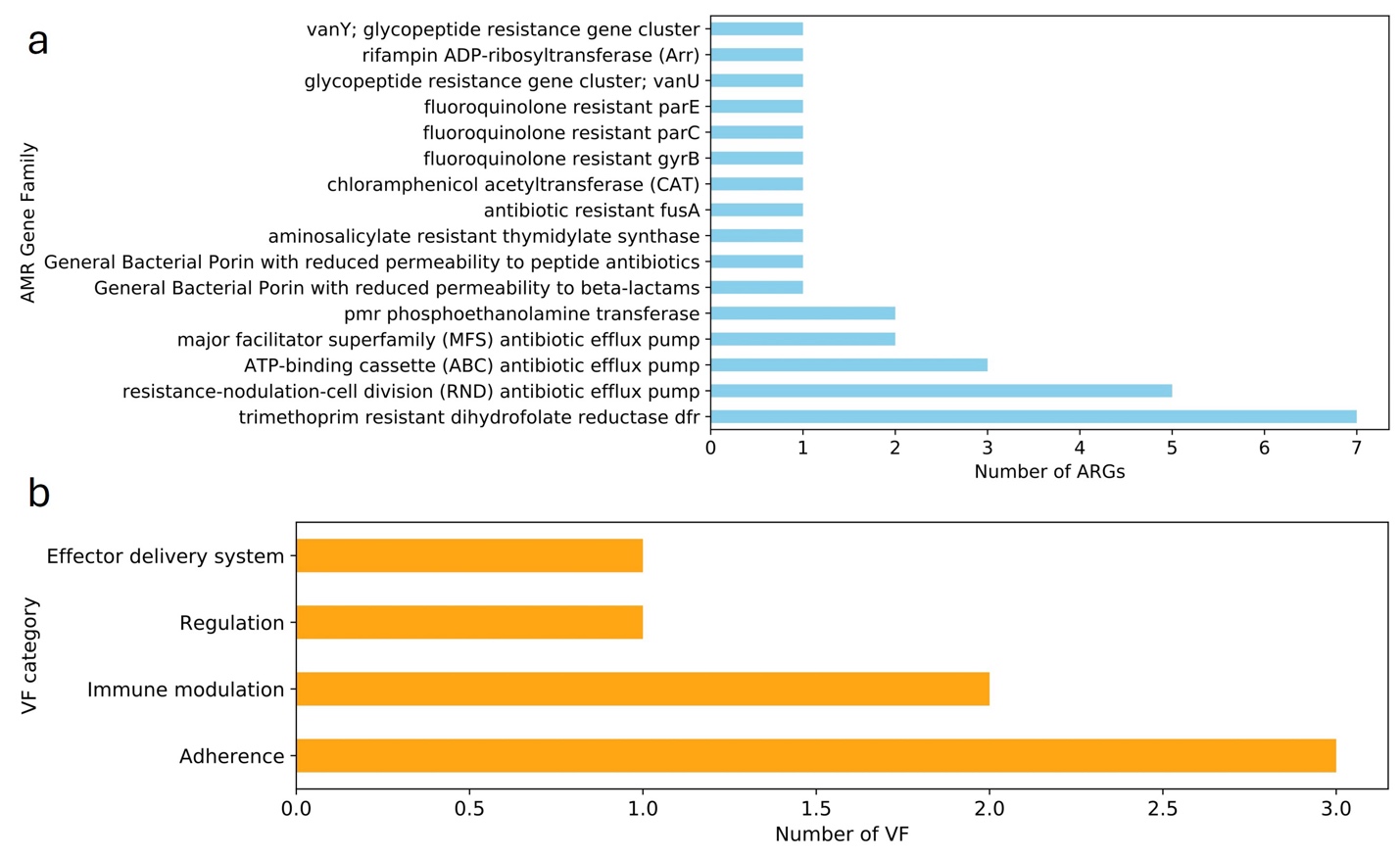


**Fig. S5** Number of antibiotic resistance genes (a) and number of virulence factor genes (b) identified in vOTU sequences.


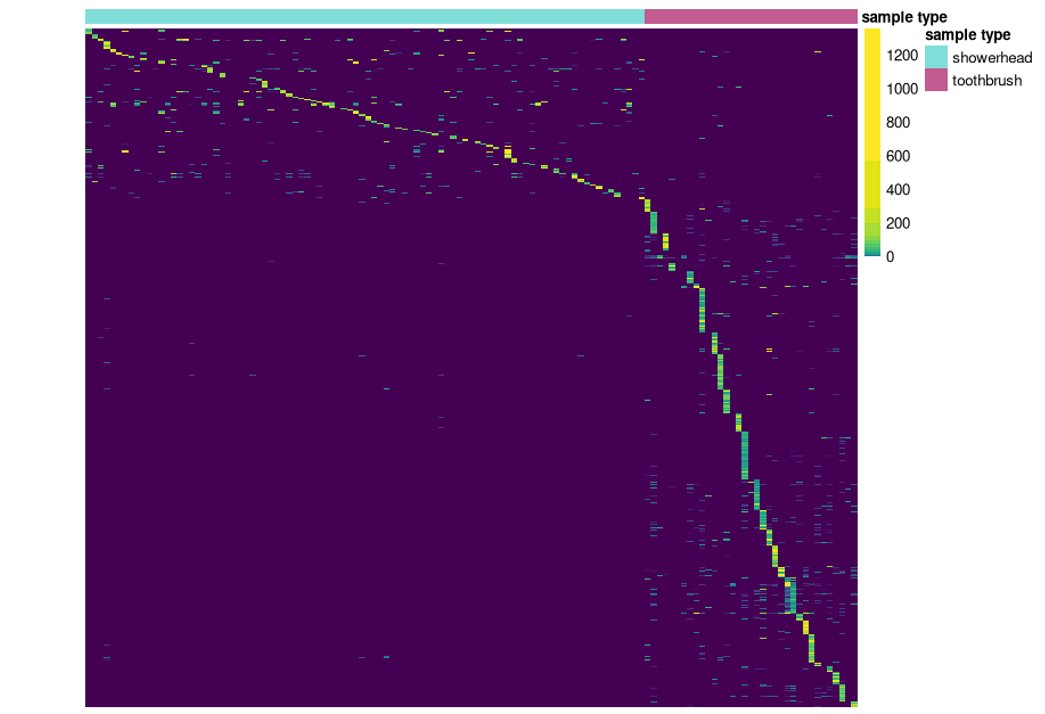
**Fig. S6** Abundance heatmap with all 614 vOTUs.


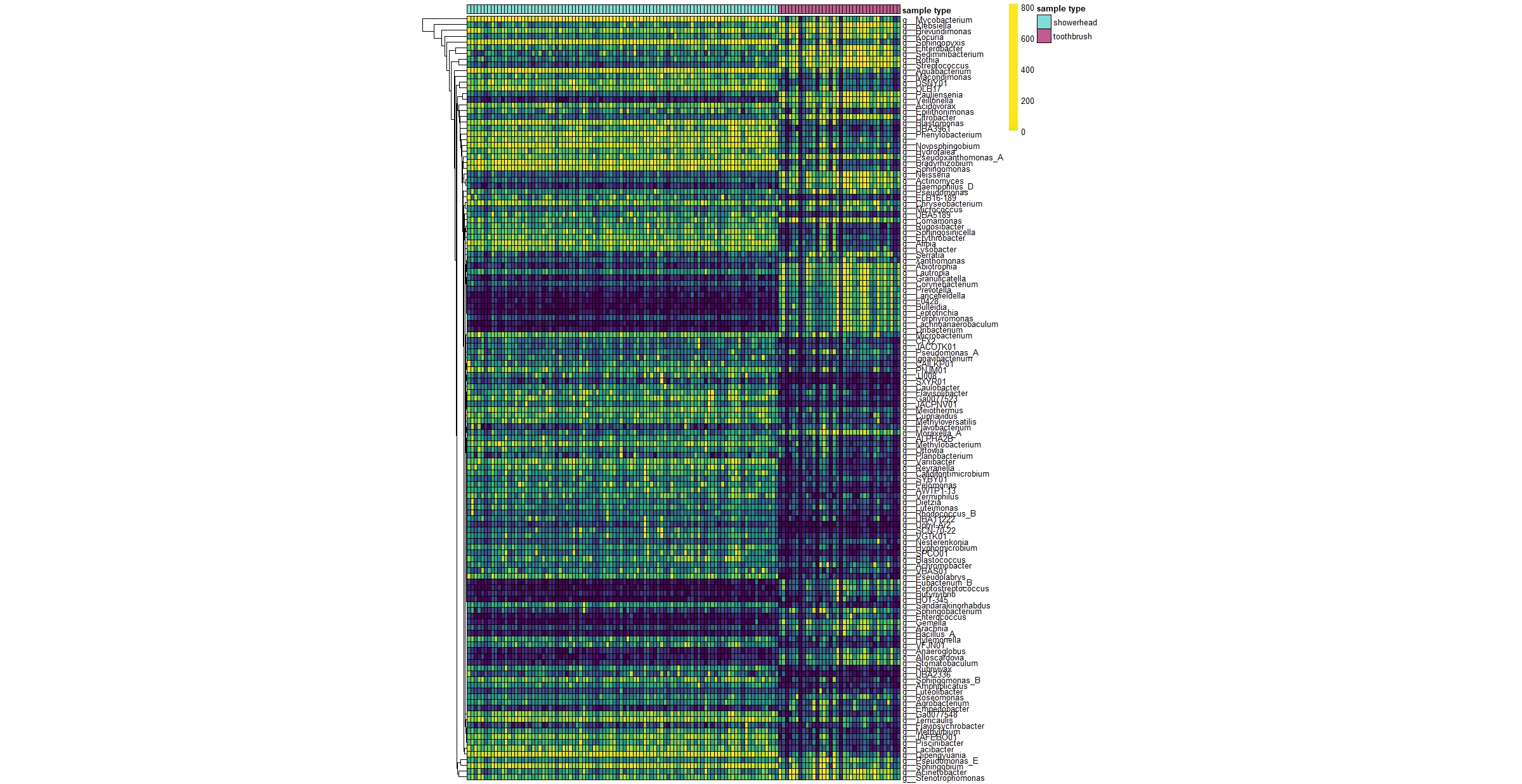


**Fig. S7** Abundance heatmap by bacterial MAG genus across all samples.
