# Supplementary figures and images for: Phage communities in household-related biofilms correlate with bacterial hosts
Source: Front Microbiomes. 2024 Oct 9;3:1396560. doi: 10.3389/frmbi.2024.1396560 (PMC12993545; doi:10.3389/frmbi.2024.1396560)

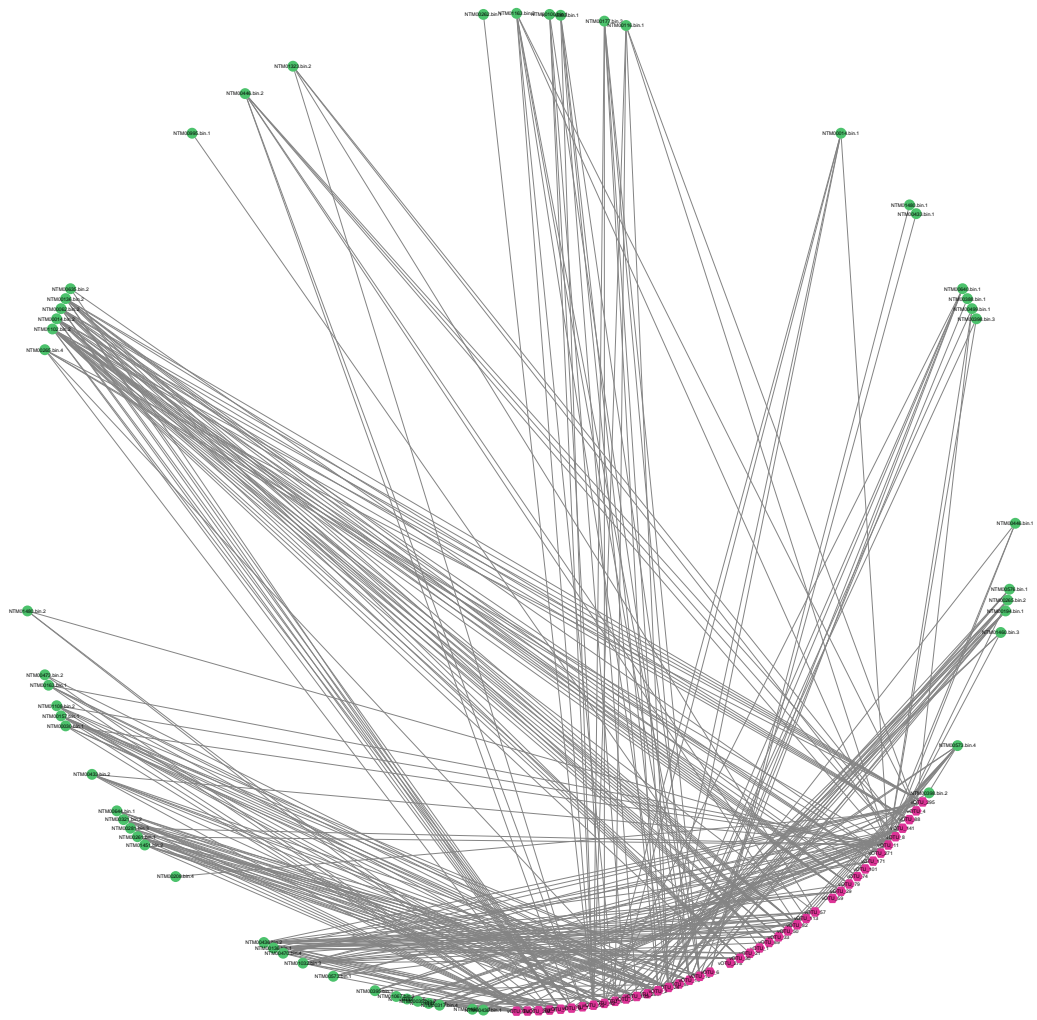

Supplement: Supplementary file 2 [file DataSheet2.pdf]
